# Investigating the impact of tumor location and size on the risk of recurrence for papillary thyroid carcinoma in the isthmus

**DOI:** 10.1002/cam4.6023

**Published:** 2023-05-03

**Authors:** Feng Zhu, Fuqiang Li, Xiaojun Xie, Yijun Wu, Weilin Wang

**Affiliations:** ^1^ The Department of Thyroid Surgery, The First Affiliated Hospital, School of Medicine Zhejiang University Hangzhou China; ^2^ The Department of Hepatobiliary and Pancreatic Surgery, The Second Affiliated Hospital Zhejiang University School of Medicine Hangzhou China

**Keywords:** prognosis, thyroid carcinoma, thyroid isthmus definition, tumor location

## Abstract

**Background:**

The purpose of the study was to investigate the ability of new parameters in distinguishing high‐risk patients of recurrence from isthmic papillary thyroid carcinomas (iPTCs).

**Methods:**

One hundred sixteen iPTC patients who underwent total thyroidectomy were identified from 3461 PTC patients from 2014 to 2019. Tumor margin to trachea midline distance (TTD), maximum tumor size (TS), and transverse diameter of trachea (TD) were measured on CT images. Cox proportional hazard models served to identify risk factors associated with recurrence‐free survival (RFS). The iPTC prognostic formula (IPF = TD/(TTD − TS) − TD/TTD) was evaluated to assess the prognosis. RFS was conducted between the different groups using the Kaplan–Meier analysis. The receiver operating characteristic (ROC) curve of each parameter was drawn to predict recurrence.

**Results:**

Central lymph node metastasis (CLNM) and extrathyroidal invasion in iPTC were 58.6% and 31.0%, respectively. Regional recurrence occurred in 16 (13.8%) patients, and no patient died or had distant metastasis. The 3‐ and 5‐year RFS of iPTC were 87.5% and 84.5%, respectively. Gender (*p* = 0.001) and PLNM (prelaryngeal lymph node metastasis) (*p* = 0.010) in cPTC (center of iPTC located between two imaginary lines perpendicular to the surface of the skin from the most lateral points of the trachea) and non‐cPTC (iPTC patients enrolled in this study excluding cPTC) groups differed significantly. A cut‐off point of tumor size >1.1 cm and IPF ≤5.57 were established to have significant differences in prognosis (*p* = 0.032 and *p* = 0.005, respectively). Multivariate analysis showed that IPF ≤5.57 was independent prognostic factor for RFS (HR: 4.415, 95%CI: 1.118–17.431, *p* = 0.034).

**Conclusion:**

This study indicated the association between IPF and RFS in iPTC patients and established new models to assess risk factors for recurrence pre‐operation. IPF ≤5.57 was significantly associated with poor RFS and might be promising parameters for predicting prognosis and surgical decision‐making pre‐operation.

## Graphical abstract

This study indicated that the previously used classification of iPTC showed no significant differences in prognosis. This is the first study to integrate the isthmic tumor position, trachea width, and tumor size to demonstrate the association between IPF and the prognosis of iPTC. IPF ≤5.57 was significantly associated with recurrence and may be promising parameters that could be used for the prognostic differentiation and preoperative surgical decision‐ making.

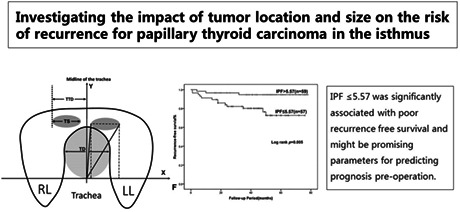

## 1 INTRODUCTION

The incidence of thyroid cancer is about 90/100,000 in China and has increased rapidly worldwide in recent years.^1^, ^2^ It has become the first malignant tumor in women under 30 years.^2^ Thyroid carcinoma in the isthmus is relatively rare because the isthmus is thinner than the lobes. However, thyroid nodules located in the isthmus had the highest risk (17.4%) of malignancy, which is higher than nodules situated in other parts of the thyroid (9.9%).^3^ Meanwhile, previous studies have demonstrated that PTCs in the isthmus are more likely to exhibit extrathyroidal invasion, central lymph node metastasis (CLNM), and multifocality than in lobes.^4^ Until now, there were no specific guidelines for the treatment of iPTC, and the extent of surgery remains controversial.

There was no precise definition for thyroid isthmus. Most of the studies did not have definite selection criteria for iPTC. Several studies used the hypothesis by drawing two imaginary lines perpendicular to the surface of the skin from the most lateral points of the trachea as the lateral margin of the isthmus. The center of the thyroid cancer is located between these two lines defined as iPTC.^5^, ^6^ Few reports use the 45° fan‐shaped area on both sides of the trachea midline.^7^ The inconsistency of the definition of isthmus may affect the selection of patients, which would influence the conclusions.

In this study, we retrospectively analyzed the clinicopathologic characteristics of iPTC. By measuring the distance from the tumor margin to the midline of the trachea, the maximum diameter of the tumor, and width of the trachea, we analyzed the influence of different locations and sizes of iPTC on the clinicopathologic characteristics and tumor recurrences, sought to find a possible model to facilitate the assessment the characteristics and prognosis of the iPTC pre‐operation.

## 2 PATIENTS AND METHODS

This retrospective study was approved by the clinical research ethics committee of The First Affiliated Hospital, School of Medicine, Zhejiang University. Written informed consent was obtained from all patients before the study.

### 2.1 Patients

A total of 3461 PTC patients underwent total thyroidectomy and bilateral cervical central lymph nodes dissection between August 2014 and October 2019 in the department of thyroid surgery of The First Affiliated Hospital, School of Medicine, Zhejiang University were analyzed retrospectively. One hundred sixteen patients (3.4%) had a dominant PTC located in the isthmus. The clinicopathological data of iPTC patients were analyzed.

Before surgery, each patient underwent ultrasound‐guided fine‐needle aspiration biopsy (FNAB) and enhanced cervical high‐resolution computed tomography (CT) imaging with a thickness of 1.25 mm. Two experienced surgeons performed all surgical operations (Dr. Wu & Dr. Xie). Thin slice CT images were exported for measurement. CT image data were collected and measured by two technical staff and reviewed by a radiologist to ensure accuracy.

The patients in the study met the following inclusion criteria: the center of PTC was in the fan‐shaped area, which the cervical vertebrae as the apex and 45° outside the midline of the trachea (MT). Patients who underwent unilateral lobectomy or isthmectomy, multifocal carcinoma, isthmic microcarcinoma less than 3 mm, secondary surgery, preoperative neck US suspected cervical lymph node metastasis (cN1), and other types of thyroid cancer were excluded.

To detect the serum levels of thyroid stimulating hormone (TSH), thyroid peroxidase antibody (TPOAb), and thyroglobulin (Tg), electrochemiluminescence assay (Cobas 601, Roche Diagnostic) was performed. The reference ranges for TSH, TPOAb, and Tg were 0.350–4.940 mIU/L, 0.00–5.61 IU/mL, and 0.00–55.00 ng/mL, respectively.

### 2.2 Image analysis

One hundred sixteen iPTC patients' cervical CT images were collected and measured. CT image of the maximum tumor diameter of the iPTC of each patient was exported. The angle of the CT images will be adjusted if the neck deflection occurs during scanning so that the midline of the trachea and cervical vertebra coincide to avoid measurement deviations. CT images were measured independently by two technicians, and the average value was calculated. A re‐measure will be taken if the results differ significantly.

**FIGURE 1:**
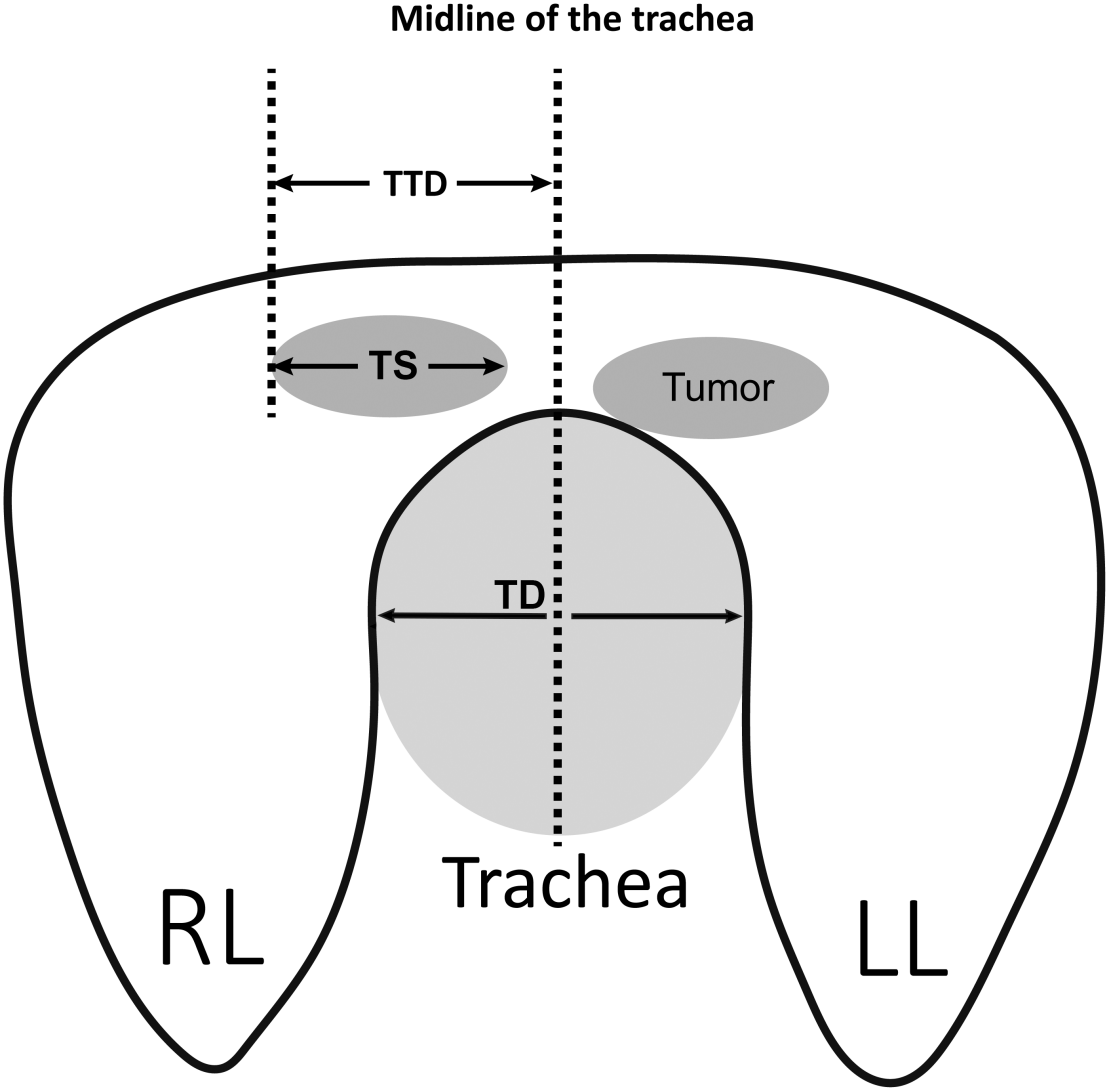
Schematic illustration of the definitions used in this study. RL, right lobe; LL, left lobe; TTD was defined as the distance of the horizontal line between the outermost line of the tumor and the midline of the trachea. This distance was subject to two conditions, first, the outermost line of the tumor must be parallel to the midline of the trachea, and second, the horizontal line reflecting the distance must be perpendicular to the midline of the trachea and outermost line of the tumor; TD, transverse diameter of trachea; TS, maximum tumor size.

TTD (RTTD: TTD on the right side & LTTD: TTD on the left side), TS, and TD were measured simultaneously on the CT image when the iPTC's diameter was largest (Figure 1). To investigate the impact of tumor size and location on clinicopathologic characteristics and prognosis, the abovementioned measurement parameters were converted to two variables: (1) TTD minuses TD/2 was less or greater than TS/2 as a basis of classification of cPTC and non‐cPTC groups; (2) IPF represents the difference between the slopes of the two straight lines from the two endpoints of the tumor transverse diameter to the origin of the coordinate system (IPF = TD/(TTD − TS) − TD/TTD. When the iPTC passes through the MT, LTTD was a negative number on the left side of the *y* axis, IPF = TD/RTTD + TD/LTTD). The coordinate system takes the MT as the *y*‐axis, and the tangent line perpendicular to the *y*‐axis and passing through the posterior edge of the trachea as the *x*‐axis. At the same time, assume that the trachea is a circle.

### 2.3 Follow‐up

All patients received thyroid‐stimulating hormone‐suppressive therapy after surgery. Postsurgical physical examinations were performed every 3–6 months. Cervical CT and FNAB were performed during a follow‐up to evaluate suspected recurrences. Reoperation was performed on patients' suspected recurrence, and postoperative pathology was confirmed.

### 2.4 Statistical analysis

Statistical analyses were performed using SPSS version 22.0 (SPSS). The results were expressed as mean ± SD. Differences between categorical variables were assessed using the Chi‐square test or Fisher exact test and continuous variables using Student's *t*‐test. Recurrence‐free survival (RFS) was analyzed between the different groups with Kaplan–Meier analysis with log‐rank test. Multivariate recurrence analysis was conducted using the cox proportional hazards regression to identify independent prognostic factors. Results were reported as hazard ratios (HR) and 95% confidence intervals (95% CI). X‐tile software (Version 3.6.1) a bio‐informatics tool produced by Robert et al.,^8^ combined with RFS calculated the optimal cut‐off points of tumor size. The probability for predicting recurrence was calculated using multivariate logistic regression analysis. Receiver operating characteristic (ROC) curve was performed to assess the recurrence based on the obtained predicted probabilities. Differences with *p* values <0.05 were defined as statistically significant.

## 3 RESULTS

Among the 3461 patients, 116 patients (3.4%) had PTC located in the isthmus. The clinicopathologic features of iPTC patients were listed in Table 1. The male‐to‐female ratio was 1:4.52, with a mean age of 43.69 years (range, 19–77) at the time of diagnosis. The mean tumor size is 1.13 ± 0.65 cm, and the proportions of ≤1 cm, 1‐2 cm, 2‐3 cm, and >3 cm were 56.9%, 36.2%, 5.2%, and 1.7%, respectively. Extrathyroidal invasion and CLNM of iPTCs were 31.0% and 58.6%, respectively. The types of extrathyroidal invasions include subhyoid muscles, trachea, and cricothyroid muscle (22.4%, 7.8%, and 0.9%, respectively). The mean distances of RTTD and LTTD were 0.88 ± 0.54 cm and 0.87 ± 0.57 cm. The mean transverse diameter of trachea was 1.56 ± 0.18 cm. IPTC on the right and left sides of the MT and across the MT were 26.7%, 19.0%, and 54.3%, respectively.

**TABLE 1:** Clinicopathologic characteristics of patients with isthmic papillary thyroid carcinomas.

Characteristic	Total (*n* = 116)
Sex	
Male, *n* (%)	20 (17.2%)
Female, *n* (%)	96 (82.8%)
Age, years, mean (range)	43.69 (19–77)
Tumor location, *n* (%)	
Right side of MT	22 (19.0%)
Across the MT	63 (54.3%)
Left side of MT	31 (26.7%)
Tumor size, cm (mean ± SD)	1.13 ± 0.65
≤1, *n* (%)	66 (56.9%)
1 to ≤2, *n* (%)	42 (36.2%)
2 to ≤3, *n* (%)	6 (5.2%)
>3, *n* (%)	2 (1.7%)
CLNM, *n* (%)	
Yes	68 (58.6%)
No	48 (41.4%)
Extrathyroidal invasion, *n* (%)	
Yes	36 (31.0%)
No	80 (69.0%)
Type of invasion, *n* (%)	
Subhyoid muscles	26 (22.4%)
Trachea	9 (7.8%)
Cricothyroid	1 (0.9%)
Recurrence, *n* (%)	
Yes	16 (13.8%)
No	100 (86.2%)
Distance to MT	
RTTD, cm (mean ± SD)	0.88 ± 0.54
LTTD, cm (mean ± SD)	0.87 ± 0.57
Trachea width, cm (mean ± SD)	1.56 ± 0.18
TG, ng/mL (mean ± SD)	54.55 ± 123.94
TPOAb, IU/mL (mean ± SD)	378.78 ± 994.56
TSH, μIU/mL (mean ± SD)	1.95 ± 3.24
Follow‐up time, months, median (range)	48.5 (1–78)

Abbreviations: CLNM, central lymph node metastases; LTTD, TTD on the left side; MT, midline of trachea; RTTD, TTD on the right side; SD, standard deviation; TG, thyroglobulin; TPOAb, thyroid peroxidase antibody; TSH, thyroid stimulating hormone; TTD, tumor margin to trachea midline distance.

The clinicopathologic features of the cPTC and non‐cPTC groups were compared (Table 2). There were no significant differences in age, CLNM, extrathyroidal invasion, thyroglobulin, thyroid stimulating hormone, and thyroid peroxidase antibodies between the cPTC and non‐cPTC groups (*p* = 0.846, *p* = 0.361, *p* = 0.439, *p* = 0.566, *p* = 0.230, and *p* = 0.621, respectively). The mean tumor size was 1.13 ± 0.68 cm in the cPTC group and 1.11 ± 0.57 cm in the non‐cPTC group (*p* = 0.859). The rate of female patients in the two groups was 89.5% and 63.3%, respectively (*p* = 0.001). The rate of PLNM (9 of 86 [10.5%] vs. 7 of 30 [23.3%]; *p* = 0.010) was significantly lower in the cPTC group than in the non‐cPTC group. However, there were no statistically significant differences in recurrence between two groups (*p* = 0.120).

**TABLE 2:** Comparison clinicopathologic characteristics between the center of iPTCs located in two lines or not.

Characteristic	cPTC (*n* = 86)	non‐cPTC (*n* = 30)	*p* value
No. of females, *n* (%)	77 (89.5%)	19 (63.3%)	**0.001**
Age (mean ± SD, years)	43.56 ± 12.13	44.07 ± 12.97	0.846
>55/≤ 55	19/67	5/25	0.528
Tumor size (mean ± SD, cm)	1.13 ± 0.68	1.11 ± 0.57	0.859
>1/≤1	36/50	14/16	0.647
PLNM, *n* (%)	9 (10.5%)	7 (23.3%)	**0.010**
CLNM, *n* (%)	48 (55.8%)	20 (66.7%)	0.361
Extrathyroidal invasion, *n* (%)	25 (29.1%)	11 (36.7%)	0.439
Recurrence, *n* (%)	9 (10.5%)	7 (23.3%)	0.120
TG (mean ± SD, ng/mL)	50.34 ± 118.55	66.14 ± 139.36	0.566
TPOAb (mean ± SD, IU/mL)	311.75 ± 930.85	566.45 ± 1151.04	0.230
TSH (mean ± SD, μIU/mL)	2.04 ± 3.75	1.70 ± 0.85	0.621

Abbreviations: CLNM, central lymph node metastases; cPTC, center of iPTC located between two imaginary lines perpendicular to the surface of the skin from the most lateral points of the trachea; iPTCs, isthmic papillary thyroid carcinomas; non‐cPTC, iPTC patients enrolled in this study excluding cPTC; PLNM, prelaryngeal lymph node metastasis; SD, standard deviation; TG, thyroglobulin; TPOAb, thyroid peroxidase antibody; TSH, thyroid stimulating hormone.

The bolded *p*‐values represent statistically significant.

**FIGURE 2:**
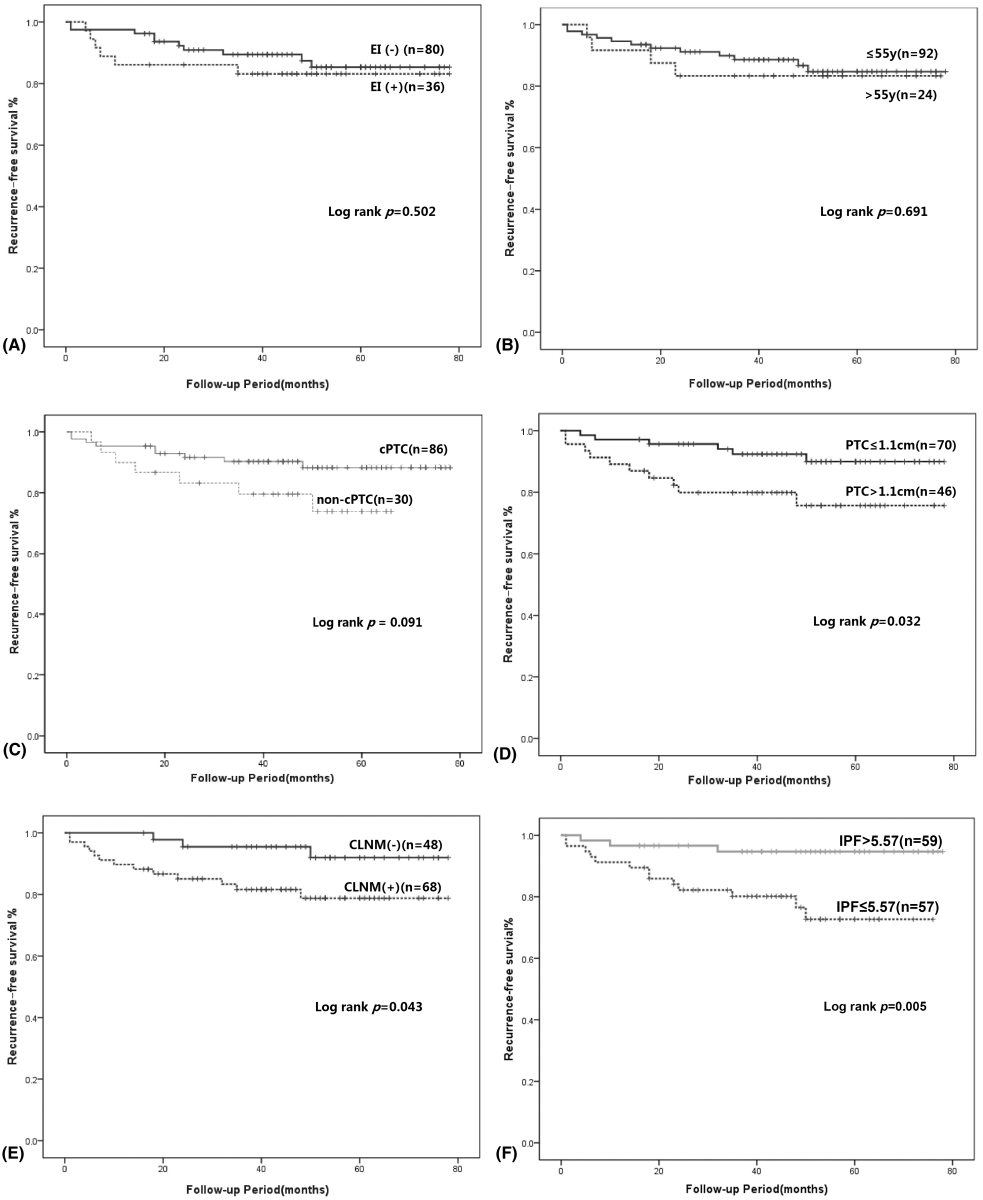
Recurrence‐free survival according to the presence of extrathyroidal invasion (EI) (A), age (≤55 years and > 55 years) (B), tumor location (cPTC and non‐cPTC) (C), tumor size (≤1.1 cm and >1.1 cm) (D) and CLNM (E) in iPTC. (F) the association between the IPF (≤ 5.57) and recurrence‐free survival in iPTC. The Kaplan–Meier method for recurrence with the log‐rank test was used for statistical comparisons. CLNM: central lymph node metastasis. TTD, tumor margin to trachea midline distance; cPTC, center of the PTC was located between two lines perpendicular to the most lateral points of the trachea; non‐cPTC, iPTC patients enrolled in this study excluding cPTC.

**FIGURE 3:**
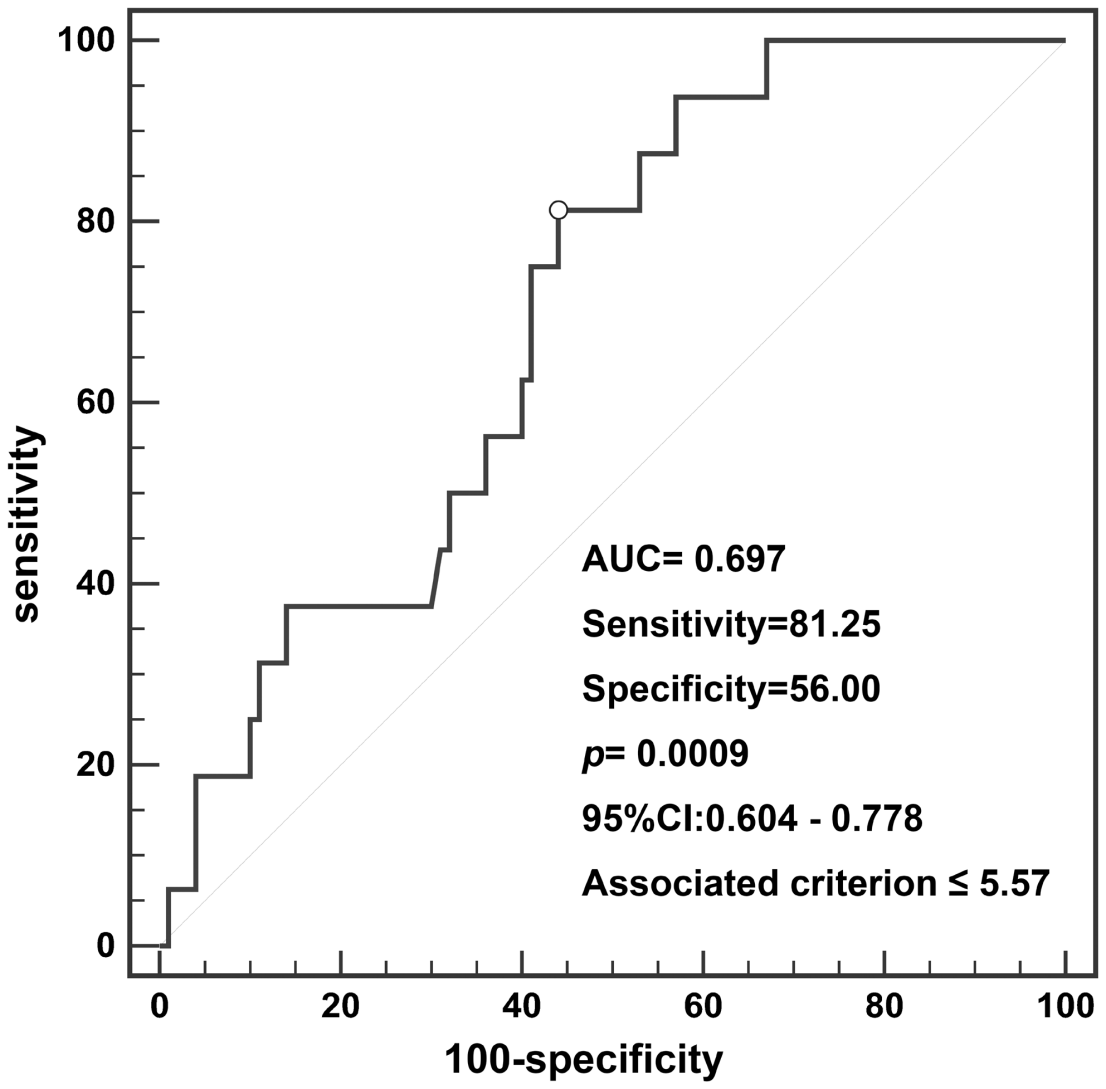
Receiver operating characteristic curve analyses of the value of IPF for predicting recurrence.

During the follow‐up period, 16 (13.8%) patients developed recurrence. The median time to recurrence after surgery was 48.5 months (range, 1–78 months). No patients died or had distant metastasis. Kaplan–Meier survival analysis with log‐rank test was performed to predict prognosis. The entire cohort's 3‐ and 5‐year RFS were 87.5% and 84.5%. There were no statistical differences for recurrence in terms of extrathyroidal invasion and age (≤ 55 years) (*p* = 0.502 and *p* = 0.691) (Figure 2A,B). There were no significant differences in prognosis between cPTC and non‐cPTC groups (*p* = 0.091) (Figure 2C). Tumor size (>1.1 cm) and CLNM have significant differences in recurrence (*p* = 0.032 and *p* = 0.043) (Figure 2D,E). Reoperative surgery was performed in patients with tumor recurrence. When the 16 patients with recurrence were compared to the 100 patients without recurrence in terms of their clinicopathological variables, they were found to have higher proportion of CLNM (*p* = 0.041) and lower IPF value (*p* = 0.029) (Table 3). ROC curve was performed to assess the recurrence based on the IPF (Figure 3). The area under the ROC curve (AUC) was 0.697 *(p* = 0.0009), which indicated that the IPF could accurately predict recurrence in iPTCs. According to Youden's index, the best cutoff value was 5.57. The sensitivity and specificity were 81.25% and 56.00%. IPF >5.57 and IPF ≤5.57 have significant differences in recurrence (*p* = 0.005) (Figure 2F).

**TABLE 3:** Clinicopathologic factors related to tumor recurrence.

Characteristic	Recurrence (*n* = 16)	No recurrence (*n* = 100)	*p* value
Age (years), mean ± SD	45.13 ± 12.94	43.46 ± 12.24	0.764
Age ≤ 55 years, *n* (%)	12 (75.0%)	80 (80.0%)	0.431
Female, *n* (%)	11 (68.8%)	85 (85.0%)	0.111
CLNM, *n* (%)	13 (81.3%)	55 (55.0%)	**0.041**
cPTC, *n* (%)	7 (43.8%)	23 (23.0%)	0.077
Extrathyroidal invasion, *n* (%)	6 (37.5%)	30 (30.0%)	0.018
Mean no. dissected nodes for CND	10.63 ± 8.22	8.96 ± 6.48	0.074
Mean no. metastasis nodes for CND	2.50 ± 2.76	2.28 ± 3.60	0.481
Ratio of CLNM	0.27 ± 0.30	0.24 ± 0.30	0.668
IPF	4.00 ± 2.62	7.88 ± 6.93	**0.029**
IPF ≤5.57, *n* (%)	13 (81.3%)	44 (44.0%)	**0.005**

Abbreviations: CLNM, Central lymph node metastases; CND, Central neck dissection; cPTC, Center of iPTC located between two imaginary lines perpendicular to the surface of the skin from the most lateral points of the trachea; IPF, IPTC prognostic formula (IPF = TD/(TTD − TS) − TD/TTD); TD, Transverse diameter of trachea; TS, Maximum tumor size; TTD, Tumor margin to trachea midline distance.

The bolded *p*‐values represent statistically significant.

Univariate analysis was conducted using the cox proportional hazards regression to identify independent prognostic factors, and found CLNM (+), tumor size (>1.1 cm) and IPF (≤5.57) were significantly associated with poor RFS. Multivariate cox regression analysis found that IPF (≤5.57) was independent prognostic factor for recurrence (HR: 4.415, 95%CI: 1.118–17.431, *p* = 0.034) (Table 4).

**TABLE 4:** Univariate and multivariate analysis of variables associated with tumor recurrence.

Variables	Univariate		Multivariate	
	HR (95% CI)	*p* value	HR (95% CI)	*p* value
Sex (vs. female)	2.274 (0.789–6.552)	0.128		
Age (vs. ≤55year)	1.257 (0.405–3.897)	0.692		
Extrathyroidal invasion (vs. no invasion)	1.412 (0.513–3.890)	0.504		
cPTC (vs. non‐cPTC)	2.313 (0.861–6.215)	0.096		
CLNM (vs. CLNM(−))	3.551 (1.010–12.481)	**0.048**	2.823 (0.716–11.120)	**0.138**
Tumor size (vs. ≤1.1 cm)	2.874 (1.043–7.916)	**0.041**	1.600 (0.488–5.243)	**0.438**
IPF (vs. >5.57)	5.515 (1.479–20.563)	**0.011**	4.415 (1.118–17.431)	**0.034**

Abbreviations: CLNM, central lymph node metastases; IPF, IPTC prognostic formula (IPF = TD/(TTD − TS) − TD/TTD); TD, transverse diameter of trachea; TS, maximum tumor size; TTD, tumor margin to trachea midline distance.

The bolded *p*‐values represent statistically significant.

## 4 DISCUSSION

The isthmus is located between the trachea and infrahyoid muscles, with an average thickness of about 3.4 ± 1.7 mm, and is slightly thicker in females.^9^ Recent studies have found that the proportion of carcinoma of thyroid nodules located in the isthmus was 11.6%–17.4%, higher than the proportion of 5.1%–9.9% in the lobes.^3^, ^10^ In previous studies, the ratio of iPTC has been reported approximately 2.2%–12.3% of all PTC in previous studies,^5^, ^11^ which was 3.4% in our study. The reason for the wide range in the proportion of iPTC may be related to the different selection criteria. All patients in this study underwent total thyroidectomy. Patients who underwent unilateral lobectomy or isthmectomy, secondary surgery, other types of thyroid cancer, multifocal carcinoma, and tumor size smaller than 3 mm were excluded.

iPTC may have a higher incidence of extrathyroidal invasion, CLNM, and multifocality than PTC in the lobes because even a small tumor abuts the trachea and the thyroid capsule.^5^, ^12^ Lee et al. observed that the rate of extrathyroidal invasion and CLNM of iPTC were 100% and 71.4%, respectively,^6^ while the results of Hahn et al. were 83.3% and 68.8%, respectively,^5^ which were much higher than those of non‐isthmic PTCs (15.5% and 27.1%, respectively).^13^ Goldfarb et al. reported that the proportion of multifocality of iPTC was 67%,^14^ which was significantly higher compared with PTCs in other sites (20%–30%).^15^ Nonetheless, some studies have shown the opposite opinion. In the study of Wang et al., the rate of extrathyroidal invasion of iPTC was 11%.^16^ In the study of Karatzas et al., the rate of CLNM was 29.6%.^17^ The rate of extrathyroidal invasion and CLNM were 31% and 58.6% in this study. Due to its unique anatomical location, thyroid isthmus has different lymphatic drainage compared to the thyroid lobe and in particular, lymphatic isthmic vessels usually drain into the prelaryngeal lymph node.^18^, ^19^ Chai et al. reported that the incidence of PLNM in iPTC was 15.2%,^20^ which was similar to the rate of 13.8% in this study. However, In a meta‐analysis conducted by Wang et al., the mean rate of PLNM in PTC was 16.2%.^21^ Therefore, whether iPTC affects the prelaryngeal lymph nodes was still controversial.

These differences may be due to the discordance in defining the thyroid isthmus. In most previous studies, the isthmus was not clearly defined. Hahn et al. identified the location of a tumor to be the isthmus by drawing two imaginary lines perpendicular to the surface of the skin from the most lateral points of the trachea as the lateral margin of the isthmus. The center of the tumor located between these two lines was defined as iPTC.^5^ Some studies used this selection criterion but did not explain the reason.^6^, ^11^, ^22^ In a recent study by Song et al., the location of the tumor was defined as the isthmus if the iPTC was totally confined within the lateral margin of the trachea.^23^ This may narrow down the iPTC. J. Seok et al. suggested using the 45° fan‐shaped area on both sides of the trachea midline. While the epicenter of the tumor located in the area without shifting to either side was defined as iPTC.^7^ Some studies did not mention the precise definition. A recent study showed that the location of iPTC was an additional risk factor, regardless of the other risk factors.^24^ The inconsistency of the definition of isthmus may influence iPTC patient choice and confuse the conclusions of clinical characteristics.

At present, the appropriate surgery for iPTC remains controversial. There have been many discussions about whether total thyroidectomy, lobectomy, or isthmusectomy were effective treatments in iPTC patients. Several surgeons recommend total thyroidectomy could be considered as an appropriate surgical treatment for iPTC regardless of tumor size because of the higher rates of extrathyroidal invasion, CLNM, and multiple foci.^4^, ^17^ However, the probability of recurrence of iPTC underwent total thyroidectomy was 0.9%–5.2% in previous studies,^7^, ^11^, ^25^ similar to the rate of 4.7% in all PTC patients undergoing total thyroidectomy. This suggested that although iPTC was associated with more aggressive features, the recurrence was similar to PTC located in lobes.^26^ According to the 2015 American Thyroid Association (ATA) guideline, both total thyroidectomy and lobectomy be considered when the tumor size of PTC between 1–4 cm without lymph node metastasis and extrathyroidal invasion. Meanwhile, no clear surgical guidelines for iPTC, and the focus of debate was mainly on how much thyroid tissue should be removed. In the study by Park et al., the 5‐year RFS of the low‐ and intermediate‐risk iPTC were 100% and 87.5%, respectively.^27^ Nixon et al. reported that the 10‐year RFS of pT1 and pT2 iPTC was 100%.^28^ Hence, the surgical strategy for iPTC was altered to be conservative recently,^28^, ^29^ and several studies have reported that thyroid isthmusectomy alone was an acceptable procedure in low‐risk iPTC.^27^, ^30^, ^31^ Sugenoya et al. advocated isthmusectomy as a suitable and reasonable surgical procedure for selected patients with small iPTC limited to the isthmus without lymph node metastasis.^32^ The purpose of the trend was to reduce unnecessarily extensive operations that can cause several surgical complications. With a median follow‐up of 48.5 months, the 5‐year RFS in our study was 86%, 16 patients (13.8%) were found locoregional recurrence, and no patient died or had a distant recurrence. The reason for worse outcomes than previous studies may be related to the variation in inclusion criteria. Therefore, the definition of iPTC may influence the surgical decision‐making and postoperative outcomes.

There were no studies on the definition of iPTC. Therefore, the definition of 45° fan‐shaped area on both sides of the MT was used for the selection criterion of iPTC in this study. Which accounts for a larger number of patients. The membranous structure of the “C”‐shaped tracheal cartilage may influence the precision of phase measurement. We try to take the intersection of the MT and cervical spine as the vertex of the 45‐degree sector. The cPTC and non‐cPTC groups were established to compare the difference of clinicopathologic features and prognosis. The rate of PLNM was significantly lower in the cPTC group than in the non‐cPTC group (10.5% vs. 23.3%). In the study of Li et al., PLNM was significantly correlated with sex, age, tumor size, and extrathyroidal invasion, and the male ratio in the PLNM (+) group was higher as compared to that in the PLNM (−) group (46.89% vs. 18.56).^33^ The study of Qi et al. demonstrated that the independent risk factors of PLNM were male, bilaterality, and located in the isthmus.^34^ In this study, more male patients in non‐cPTC group compared to the cPTC group (36.6% vs. 10.5%), which might be the reason for the higher proportion of PLNM. There were no statistically significant differences in age, CLNM, extrathyroidal invasion, tumor size, and recurrence between two groups. At the same time, the iPTC patients were divided into three groups: right of the MT group, left of the MT group and across the MT group. Three groups had no significant differences except for rate of PLNM (1 of 22 in the right MT group, 4.5%; 8 of 31 in the left MT group, 25.8%; 7 of 63 in the across MT group, 11.1%; *p* = 0.044). These findings indicated that the classification of iPTC by the lateral margin of the trachea might narrow the scope of patient inclusion. Meanwhile, differentiating the location of iPTC by MT alone may not fully reveal the risk factor of prognosis. The purpose of this study was to analyze a possible convenient model to differentiate iPTC and to evaluate the prognosis of iPTC.

Our results indicated that the previously used classification of iPTC showed no significant differences in prognosis. Since iPTC is a tumor type defined by location, a stable coordinate system could be established with the relatively fixed bone structure around the thyroid. Using this coordinate system to measure parameters such as tumor location and the transverse diameter of trachea, we want to find a way to redefine isthmic cancer. Therefore, we have established predictive models to assess risk factors for recurrence. We assume that the trachea is a circle, with the midline of the trachea as the *y*‐axis, and a line perpendicular to the *y*‐axis tangent to the posterior edge of the trachea as the *x*‐axis to establish a coordinate system. The IPF represents the difference between the slopes of the two straight lines from the two endpoints of the tumor transverse diameter to the origin of the coordinate system (IPF = TD/(TTD − TS) − TD/TTD). The univariate cox regression analysis indicated CLNM (+), tumor size (>1.1 cm) and IPF (≤5.57) were significantly associated with poor RFS. Multivariate analysis indicated that IPF ≤ 5.57 was independent prognostic factor for recurrence. It may be a convenient model for predicting the recurrence and evaluating the boundaries of surgery.

The present study had several limitations. First, the sample size of the iPTC was relatively small. Meanwhile, although carefully measured, the measurement bias should not be ignored. Studies with larger sample sizes and more accurate results are needed to confirm our findings. Second, the inclusion criterion in this study expands the scope of iPTC, which may affect the results. Finally, because of the complexity of iPTC location, the IPF model cannot fully explain all iPTCs. For example, when the iPTC was located on the side of the MT but infinitely close to the MT, regardless of the tumor size, the IPF >5.57, which was not consistent with the actual situation. Thus, further investigation should be explored in the future.

## 5 CONCLUSION

Our results indicated that the previously used classification of iPTC showed no significant differences in prognosis. This is the first study to integrate the isthmic tumor position, trachea width, and tumor size to demonstrate the association between IPF and the prognosis of iPTC. IPF ≤5.57 was significantly associated with recurrence and may be promising parameters that could be used for the prognostic differentiation and preoperative surgical decision‐making.

## AUTHOR CONTRIBUTIONS

**Feng Zhu:** Conceptualization (lead); data curation (lead); formal analysis (lead); investigation (lead); software (lead); writing – original draft (lead). **FuQiang Li:** Data curation (equal); investigation (equal); methodology (equal); resources (equal); writing – original draft (equal). **XiaoJun Xie:** Data curation (equal); formal analysis (equal); investigation (equal); methodology (equal); software (equal); writing – original draft (equal). **YiJun Wu:** Conceptualization (lead); data curation (equal); funding acquisition (equal); investigation (equal); methodology (equal); project administration (lead); supervision (lead); validation (lead); writing – review and editing (lead). **Weilin Wang:** Conceptualization (lead); funding acquisition (lead); investigation (lead); methodology (lead); project administration (lead); supervision (lead); validation (lead); writing – review and editing (lead).

## FUNDING INFORMATION

This work was supported by Zhejiang medical science and technology projects [2022KY786].

## CONFLICT OF INTEREST STATEMENT

No competing financial interests exist.

## Data Availability

All data included in this study are available upon request by contact with the corresponding author.
